# Searching for resistance genes to *Bursaphelenchus xylophilus* using high throughput screening

**DOI:** 10.1186/1471-2164-13-599

**Published:** 2012-11-07

**Authors:** Carla S Santos, Miguel Pinheiro, Ana I Silva, Conceição Egas, Marta W Vasconcelos

**Affiliations:** 1 CBQF – Centro de Biotecnologia e Química Fina, Escola Superior de Biotecnologia, Centro Regional do Porto da Universidade Católica Portuguesa, Rua Dr. António Bernardino Almeida, Porto, 4200-072, Portugal; 2 Bioinformatics Unit, Biocant, Parque Tecnológico de Cantanhede, Núcleo 04, Lote 03, Cantanhede, 3060-197, Portugal; 3 Advanced Services Unit, Biocant, Parque Tecnológico de Cantanhede, Núcleo 04, Lote 03, Cantanhede, 3060-197, Portugal

## Abstract

**Background:**

Pine wilt disease (PWD), caused by the pinewood nematode (PWN; *Bursaphelenchus xylophilus)*, damages and kills pine trees and is causing serious economic damage worldwide. Although the ecological mechanism of infestation is well described, the plant’s molecular response to the pathogen is not well known. This is due mainly to the lack of genomic information and the complexity of the disease. High throughput sequencing is now an efficient approach for detecting the expression of genes in non-model organisms, thus providing valuable information in spite of the lack of the genome sequence. In an attempt to unravel genes potentially involved in the pine defense against the pathogen, we hereby report the high throughput comparative sequence analysis of infested and non-infested stems of *Pinus pinaster* (very susceptible to PWN) and *Pinus pinea* (less susceptible to PWN).

**Results:**

Four cDNA libraries from infested and non-infested stems of *P. pinaster* and *P. pinea* were sequenced in a full 454 GS FLX run, producing a total of 2,083,698 reads. The putative amino acid sequences encoded by the assembled transcripts were annotated according to Gene Ontology, to assign *Pinus* contigs into Biological Processes, Cellular Components and Molecular Functions categories. Most of the annotated transcripts corresponded to *Picea* genes-25.4-39.7%, whereas a smaller percentage, matched *Pinus* genes, 1.8-12.8%, probably a consequence of more public genomic information available for *Picea* than for *Pinus*. The comparative transcriptome analysis showed that when *P. pinaster* was infested with PWN, the genes malate dehydrogenase, ABA, water deficit stress related genes and PAR1 were highly expressed, while in PWN-infested *P. pinea,* the highly expressed genes were ricin B-related lectin, and genes belonging to the SNARE and high mobility group families. Quantitative PCR experiments confirmed the differential gene expression between the two pine species.

**Conclusions:**

Defense-related genes triggered by nematode infestation were detected in both *P. pinaster* and *P. pinea* transcriptomes utilizing 454 pyrosequencing technology. *P. pinaster* showed higher abundance of genes related to transcriptional regulation, terpenoid secondary metabolism (including some with nematicidal activity) and pathogen attack. *P. pinea* showed higher abundance of genes related to oxidative stress and higher levels of expression in general of stress responsive genes. This study provides essential information about the molecular defense mechanisms utilized by *P. pinaster* and *P. pinea* against PWN infestation and contributes to a better understanding of PWD.

## Background

PWD is caused by the pine wood nematode (PWN) *Bursaphelenchus xylophilus* (Steiner & Buhrer) Nickle. The disease affects connifers around the world, particularly in Canada, China, Japan, Korea, Mexico, Portugal and USA [1] causing serious economic damage in the affected areas.

*Pinus* spp. are the main hosts of PWN and in Portugal *P. pinaster* and *P. pinea* are the predominant pine species. Whilst the first species is extremely affected by PWN, the second appears to be less susceptible [2]. PWN can infect and kill *P. pinea*, however the disease develops slower than in *P. pinaster*[3].

The PWN is conveyd to pine trees by the longhorn beetles of the *Monochamus* spp. [4]. When the insect vector feeds on pine twigs, the nematodes are injected into the tree through the beetles’ feeding wounds [5]. After invasion, the nematodes move rapidly through the resin canals of the xylem and cortex, feeding on epithelial cells, and causing blockage of the vascular function and cavitation, alongside with water transport disruption [4]. This results in decreased water potential, cessation of resin exudation, discoloration of needles and, ultimately, tree death [6,7].

Several hypotheses have been proposed about the PWN pathogenic mechanism, however a complete understanding of the process has not been achieved [8]. Plant cell wall degrading enzymes and expansins are some of the proteins thought to be important in the nematode parasitic process [9]. And contrary to what was initially thought, PWN is not the only etiologic agent of the disease; it is possible that bacteria adherent to the body wall of PWN may contribute to the pathogenesis of the disease [2,10].

Publicly available databases have scarce information on conifer genes and 30% of these genes have little or no sequence similarity to plant genes of known function [11]. Useful initiatives have been created such as EuroPineDB, that aims at providing a high coverage database for maritime pine (*P. pinaster*) transcriptome genes [11]. Different technologies have given us some insight regarding the pine genome and its response to biotic and abiotic stresses. A few examples include: 1) single nucleotide polymorphism genotyped using GoldenGate assay, where a consensus map was created for maritime pine [12]; 2) microarray technology, that identified 2,445 differentially expressed genes that were responsive to severe drought stress in roots of loblolly pine [13]; 3) LongSAGE technique, that provided a total of 20,818 tags, from which 38 were differentially expressed in the resistant Japanese black pine and 25 in non-resistant pine [14]; 4) and suppression subtractive hybridization, showing the up-regulation of stress response and defense related genes by pine wood nematode infestation [15,16].

High throughput 454 pyrosequencing is a powerful method for whole genome transcriptome analysis and gene discovery, and has been utilized for *P. contorta* transcriptome characterization and marker development [17]. 454 GS FLX (Roche) platform is specially useful in characterizing genetic variability of single highly polymorfic and multi-copy genes, for which many very different variants may co-occur within individuals [18].

We studied *Pinus* spp. at a transcriptional level for a better understanding of the plant’s molecular response to nematode infestation. Here, we report the 454 pyrosequencing of cDNAs from two pine species: one that exhibits susceptibility to PWN (*P. pinaster*) and the other that is less susceptible (*P. pinea*). More than 2,000,000 reads were assembled, genes potentially up-regulated by PWN infestation were identified, and the differential expression of twenty of these genes was confirmed by quantitative real time polymerase chain reation (qPCR). A total of 1,224,042 and 859,656 reads from *P. pinaster* and *P. pinea*, respectively, were added to the Sequence Read Archive (SRA), significantly increasing the available genomic information for *Pinus* spp.

## Results and discussion

### Sequence analysis

**Figure 1:**
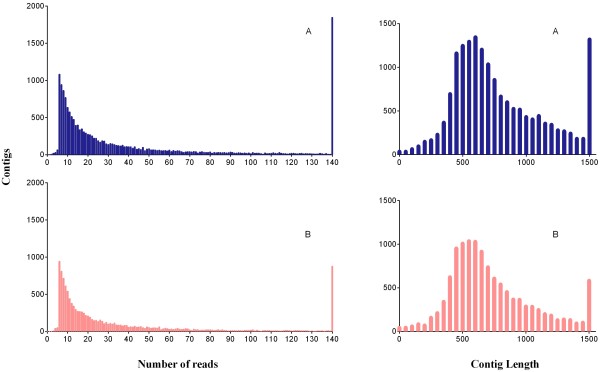
**Transcriptome assembly of PWN-infested *P. pinaster *and *P. pinea*.** Distribution of number of read per contig (left panel graphics) and size distribution of 454 sequences after assembly (right panel graphics) in normalized library. **A**) Infested *P. pinaster*; **B**) Infested *P. pinea*. The number of contigs presenting the indicated amount of reads is plotted as a histogram.

A cDNA library was constructed from RNA of pine stem tissues from *P. pinaster* and *P. pinea* inoculated with *B. xylophilus* and from uninfested controls. Pyrosequencing of the four cDNA libraries generated a total of 1,393,970 reads, with an average lengh of 320 bp. Specifically, we obtained 450,053 reads differentially expressed by *P. pinaster* infested with nematode, which assembled into 12,157 contigs; 375,168 reads for *P. pinaster* control, assembled into 8,808 contigs; 342,141 reads for *P. pinea* infested with nematode, assembled into 9,555 contigs; and 226,608 quality reads for *P. pinea* control, that were assembled into 4,175 contigs. This data is presented in Table 1. No singletons were obtained when the samples were compared, and the distribution of contig length and EST assembly by contig is shown in Figure 1, for the four samples.

**Table 1:** Summary of assembly and EST data

	**Infested* P. pinaster***	**Control* P. pinaster***	**Infested* P. pinea***	**Control* P. pinea***
No. of Reads	450,053	375,168	342,141	226,608
Total Bases	145,356,992	121,441,000	111,032,000	70,672,704
Average read length after trim quality	322	323	324	311
No. of contigs	12,157	8,808	9,555	4,175
Average contig length	806	738	783	636
Range contig length	32-3,968	12-4,031	38-4,665	11-2,828
No. of Contigs with 2 reads	8	0	0	0
No. of Contigs with > 2 reads	12,149	8,808	9,555	4,175
Contigs with BLASTx matches (E-value ≤ 10^-6^)	531	422	521	207
*Contigs with BLASTx matches (E-value ≤ 10^-2^)	3,532	2,169	2,339	1,436
Contigs determined by ESTscan	511	435	413	424
Total no. of transcripts	13,003	9,250	9,968	5,516

*Contigs without BLASTx matches at an E-value cut-off of 10^-6^ were queried again with BLASTx with an E-value cut-off of 10^-2^.

### Fuctional annotation

**Figure 2:**
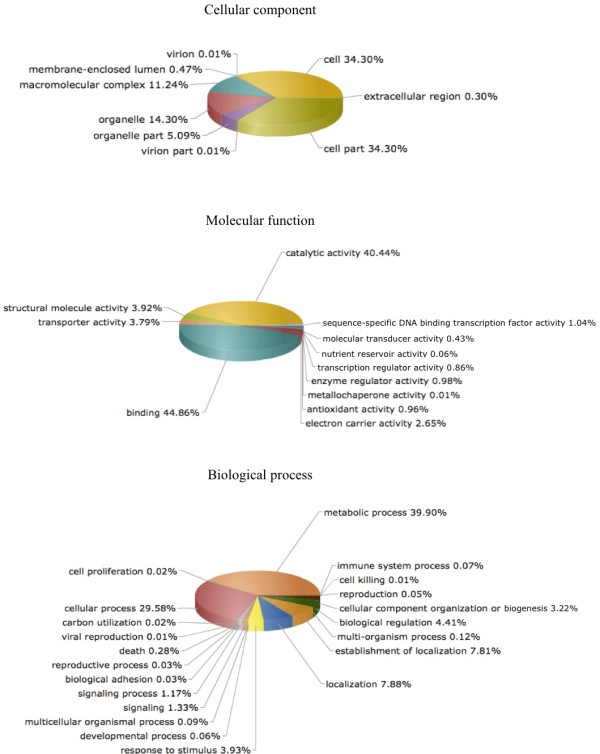
**Classification of the annotated amino acid sequences for *P. pinaster *inoculated with PWN.** The 454 sequencing data from the four samples in study were compiled, and amino acid sequences were grouped into different functional sub-categories within the Cellular component, Molecular function and Biological Process Gene Ontology (GO) organizing principles.

To annotate the transcripts, the putative frames were queried against the InterPro database of protein families and functional domains http://www.ebi.ac.uk/InterPro[19,20], and additionally annotated with GO terms, to assign *Pinus* contigs into the major GO categories (Figure 2), namely, Biological Processes, Cellular Components and Molecular Functions in a species-independent manner [21]. As the general result for these analyses was similar for all samples, an example is represented in Figure 2, namely, *P. pinaster* infested with nematode. Within the Biological Process, 29.37% and 49.36% of assignments corresponded to “Cellular Process” (GO:0008152) and “Metabolic Process” (GO:0009987) respectively, followed by the “Localization” (GO:0051179, 8.49%) and “Establishment of Localization” (GO:0051234, 8.40%) GO categories. Furthermore, the matches of Molecular Function terms were most prevalent within the “Binding” (GO:0005488, 48.84%) and “Catalytic Activity” (GO:0003824, 36.86%) category, followed by the categories “Structural Molecule Activity” (GO:0005198, 3.52%) and “Transporter Activity” (GO:0005215, 3.62%). Finally, for the Cellular Component GO the most evident matches were within the “Cell Part” (GO:0044464, 34.72%) and “Cell” (GO:0005623, 34.72%) terms, followed by “Organelle” (GO:0043226, 13.33%) and “Macromolecular Complex” (GO:0032991, 10.76%). Together, these GO classes accounted for most of the assignable transcripts, and may represent a general gene expression profile signature for *Pinus* spp.

**Figure 3:**
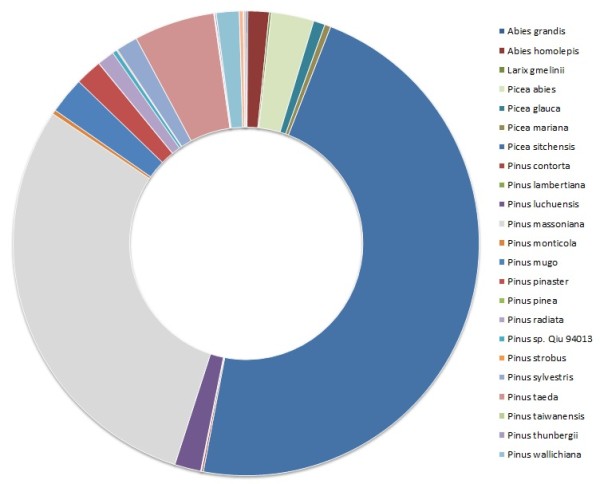
**Taxonomical analysis of the annotated sequences.** The 454 sequencing data from the four samples in study were compiled, subjected to MG-RAST analyses and the major categories are represented. Color shading of the family names indicates class membership.

Because PWD is a complex disease involving organisms of different taxons (plant, nematode and bacteria) a quantitative insight into the microbial population of the samples was conducted. For this, the taxonomical affiliation of the annotated sequences was analysed using MG-RAST [22] (Figure 3). About 50% of the sequences for each sample did not correspond to known genes in the SEED database. Remaining sequences binned to Eukaryota and, as expected, ‘Plantae’ was the Kingdom with more related sequences, correponding to 89.1% to 96.5% of the sequences (Table 2). Only 1.8% to 12.8% corresponded to *Pinus* spp. sequences, which reflects the scarce available information in public databases. As there is more genomic information in public databases available for *Picea* spp., a range of 25.4-39.8% of the ‘Plantae’ sequences belonged to this category (Table 2). Interestingly, *P. pinea* control sample was the one with the higher percentage of *Pinus* spp. sequences compared to the other samples (Table 2).

**Table 2:** Taxonomic distribution of the assembled data (percentage)

	**Eukaryota**			**Other**
	***Plantae***			
	***Pinus *spp.**	***Picea *spp.**	**Not id**	
Infested *P. pinaster*	1.8	39.0	55.7	3.5
Control *P. pinaster*	2.7	37.8	52.6	6,9
Infested *P. pinea*	1.9	39.8	47.4	10.9
Control *P. pinea*	12.8	25.4	52.1	9.7

‘Not id’ represents the percentage of sequences that had hits in databases but couldn’t be identified (unknown sequences).

### Comparing *P. pinea* and *P. pinaster* molecular responses to nematode infection

Plants have evolved a complex network of defense responses often associated with a localized response, where defenses are systemically induced in remote parts of the plant in a process known as systemic acquired resistance [23]. These are usually stimulated by incompatible interactions between a pathogen and a resistant or nonhost plant and result in two distint types of hypersensitive reaction (HR): type I, which does not produce any visible symptoms and type II, that results in rapid and localized necrotic HR [24], often eliciting *de novo* gene expression to acquire disease resistance.

**Figure 4:**
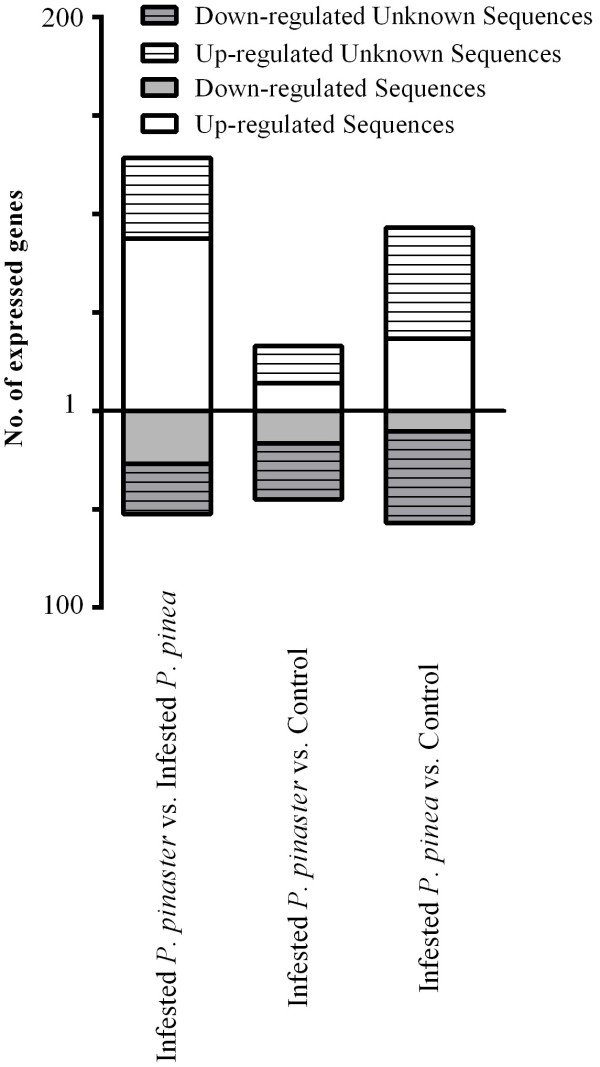
**Differentially expressed genes.** The up and down regulated genes in PWN infested *P. pinaster* and *P. pinea* are represented. Data was pooled and a ratio of the number of reads for each differentially expressed gene was calculated for each comparison. Ratios >1 were considered to be up-regulated for the numerator sample and <1, down-regulated.

To identify the participants in PWD response, the most represented genes in each sample were identified and the number of up and down regulated genes were analysed (Figure 4). In response to infestation *P. pinaster* differentially expressed 156 genes while the number of such genes in *P. pinea* was 300. When comparing between PWN infested *P. pinaster* with *P. pinea,* 257 genes had altered their altered expression levels and in the reverse comparison 105 genes were detected. Also, the expression varied between control treatments, which indicated that they were expressing different genes (data not represented). This differential expression was also observed in other studies on the effect of *B. xylophilus* 24 h after innoculation in susceptible and resistant pines [15]. There was a high percentage (around 53%) of unknown sequences that were differentially expressed – this fact could stem from the low genomic information available for *Pinus* spp. Also, the contigs without any homology may correspond to novel or diverged amino acid coding sequences, or could represent mostly 3’ or 5’ untranslated regions (UTRs), lacking protein matches as they are non-coding (Table 1).

When the infested samples were compared against the controls, both presented a similar number of down-regulated genes, 21 by *P. pinea* and 33 by *P. pinaster*, but *P. pinea* up-regulated more than double the number of genes when compared to *P. pinaster*, which supports the hypothesis that these species respond differently to the nematode infestation.

When comparing both infested samples, *P. pinaster* was the species with higher number of up-regulated genes, suggesting that, although *P. pinea* had a stronger reaction to the infestation, it differentially expressed less genes when compared to *P. pinaster* (Figure 4).

**Figure 5:**
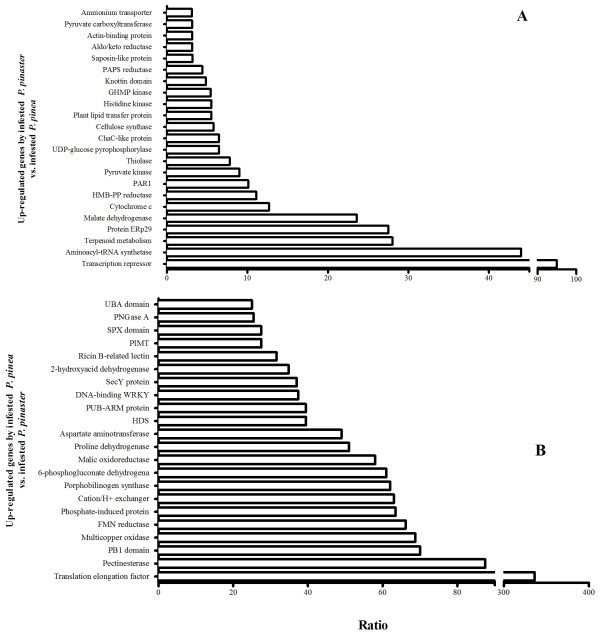
**Up-regulated genes in (A) infested *P. pinaster *compared to infested *P. pinea *and (B) infested *P. pinea *compared to infested *P. pinaster*.** Legend: PAPS - phosphoadenosine phosphosulphate; PAR1 - photoassimilate-responsive protein; HMB-PP - (E)-4-hydroxy-3-methylbut-2-enyl diphosphate; PNGase - peptide-N4-(N-acetyl-beta-glucosaminyl)asparagine amidase; PIMT - protein L-isoaspartyl (D-aspartyl) *O*-methyltransferase; HDS - 1-hydroxy-2-methyl-2-(*E*)-butenyl 4-diphosphate synthase; FMN - flavin mononucleotide. Due to the large number of up regulated sequences, only the genes with a ratio of expression higher than 3 (in panel **A**) and 25 (in panel **B**) could be represented.

Due to the differential susceptibility to the PWN, it is interesting to compare the genes expressed by both *P. pinaster* and *P. pinea* when subjected to PWN infestation. Figure 5A shows the up-regulated genes in PWN-infested *P. pinaster* when compared with PWN-infested *P. pinea*. The genes more expressed by *P. pinaster* were a transcription repressor and a translation machinery component, aminoacyl-tRNA synthetase. Transcriptional regulators are key factors in the expression of specific genes and ensure the cellular responses to internal and external stimuli [25] and thus the expression of factors related to protein synthesis could be involved in the activation of defense genes in response to the nematode attack. A ERp29 protein was also up-regulated, and this is an endoplasmic reticulum stress-inducible protein, that is activated by the accumulation of transport-incompetent, misfolded and/or underglycosylated secretory proteins [26], again related to protein regulation.

Two component signaling elements have already been found to be present in *A. thaliana* and in rice, and here a possible histidine kinase was identified. These type of proteins are associated with signal transduction mediation in multiple pathways, acting like the hormones cytokinin and ethylene [27].

As already mentioned in the Background section, the main symptom of the disease – wilting of leaves, that ultimately leads to tree death - is caused by a decrease in water potential in *B. xylophilus* infested stems [28]. When water conduction is disrupted, xylem tracheids fill with air and oleoresin due to the resulting cavitation [29]. The cavitation becomes permanent once tracheids are refilled with hydrophobic terpenoids synthesized by injured parenchyma cells [8]. Therefore, it is understandable why terpene metabolism related proteins, like (E)-4-hydroxy-3-methylbut-2-enyl diphosphate (HMB-PP) reductase and thiolase like protein, both involved in terpenoid synthesis, were differentially expressed by infested *P. pinaster* (Figure 5A) [30,31]. Subsequently, as the water potential decreases, pine trees suffer severe oxidative stress and here, likewise other PWD-related studies [16,32], several oxidative-related genes were found, namely, a cytochrome c, found in the oxidation of phenolic elements in cell wall polymers under biotic stress, that has been associated with nematode infection in other studies [32] and an aldo/keto reductase, a member of NADPH-dependent oxidoreductases, that intervenes in the elimination of reactive oxygen species produced by plant cells after suffering from a great amount of stress [33].

Another symptom caused by PWN infection is the enhancement of plants’ respiration and oxidative stress [28]. A possible malate dehydrogenase (MDH) was found to be over-expressed by infested *P. pinaster*. MDH is responsible for the interconversion of malate and oxaloacetate, regulating respiratory rate in plants [34], which may be related to the disease.

Nematodes feed off young differentiating phloem fibers and xylem ray parenchyma cells [29]. A cellulose synthase was up-regulated in infested *P. pinaster.* This enzyme is essential for primary and secondary cell wall biosynthesis [35], and could be recruited to repair wood formation induced by nematode feeding.

Interestingly, several plant defense related genes were also up-regulated by *P. pinaster* in response to the infestation. These included: a probable photoassimilate-responsive protein (PAR1) that displays features similar to pathogenesis-related proteins [36]; a putative plant lipid transfer protein (LTP), that may be involved in pathogen-defense reactions via inhibition of bacterial and fungal growth [37]; sugar related proteins - like pyruvate-related proteins, GHMP kinase and a UDP-glucose pyrophosphorylase [38-40]. These genes have been found overexpressed after pathogen infection and, in *Arabidopsis thaliana*, the expression of sugar transport proteins can be induced by wounding and pathogen attack, altering cell wall dynamics [41]; a phosphoadenosine phosphosulphate (PAPS) reductase, mainly involved in sulphate assimilation, that may contribute to plant defense, since S-containing secondary metabolites work against pathogens and herbivores [42]; and a sequence belonging to the saposin-like protein family that participates in the plant defense mechanism against fungal pathogens by membrane permeabilization [43].

In a recent study conducted in *P. thumbergii* defense response genes, an antimicrobial peptide, salycilic acid-responsive genes and jasmonic acid/ethylene-responsive genes were induced more quickly and to a higher level in susceptible than in resistant trees [15]. These gene classes were not the ones found to be more highly expressed by susceptible *P. pinaster*, possibly pointing out to a species-specific response in disease susceptibility amongst pine trees.

Perhaps the most helpful information when aiming at identifying resistance genes to the PWN derived from the analysis of the genes expressed by PWN-infested *P. pinea* (less susceptible to PWN) when compared with PWN-infested *P. pinaster*. This data is shown in Figure 5B. PWN-infested *P. pinea* had higher expression levels in general, and some of the most interesting findings included a plant disease resistance protein, which was not found to be expressed by *P. pinaster* and a ricin B-related lectin. Plant lectins have already been indicated as participants in the general defense against a multitude of plant pathogens, including nematodes [44].

The oxidative stress related multicopper oxidase, flavin mononucleotide (FMN) reductase and 6-phosphogluconate dehydrogenase [32,45,46] were all up-regulated and these proteins have a crucial role in PWD since, as previously mentioned, they are believed to play an importante role in the maintenance of intracellular redox balance and in stress response/tolerance in plants. Particularly, FMN reductase has already been identified in previous studies in our lab as possibly related to *B. xylophilus* infection [16]. Also, a phox/Bem1 (PB1) domain was found to be more represented by infested *P. pinea* (Figure 5B) and this domain is usually found in signaling proteins including oxidases and cytosolic factors [47] and a 2-hydroxyacid dehydrogenase, that is associated with 3-phosphoglycerate dehydrogenase and may play a role in the oxidation-reduction process [48].

The malic enzyme [49] and proline dehydrogenase are also involved in oxidative stress, and are believed to play an important role in plant defence.The second one was recently found in *Arabidopsis* to affect cell death and disease resistance against biotic stress by altering cellular redox state, besides other mechanisms [50].

The most up-regulated genes in infested *P. pinea* were a possible translation elongation factor, mainly involved in protein synthesis and in the regulation of different cellular processes [51], and the defense related protein pectinesterase, that belongs to a group of methyl jasmonate inducible pathogenesis-related proteins and has been correlated to cell wall extension (here justified by the need to replace the nematode feeding-damaged cell walls) and microbial pathogens inhibition [52,53]. As pointed out by others, up-regulation of cell wall-related genes contributing to the strength of cell walls would be a very effective defense against PWN infection, because these events may restrict PWN migration [15].

Other defense related proteins were differentially expressed by PWN infested *P. pinea*, like a plant U-box (PUB) protein and a WRKY protein. The first, involved in ubiquination, usually carries tandem armadillo repeats (PUB-ARM proteins) in eukaryotes. PUB-ARM proteins were identified as part of the pathogen response in tobacco and *Arabidopsis*[54,55]. The second, are transcriptionally inducible upon plathogen infection and other defense-related stimuli and, although this may not be true for all WRKY genes, the overexpression (for example) of *AtWRKY18* was shown to activate pathogenesis-related genes and to enhance resistance to certain pathogens [25,56]. Another hit possibly involved in ubiquination was detected, a UBA domain (Figure 5B). In plants, ubiquitinated proteins were described to regulate, besides germination and flowering, cell cycle and processes of response to the majority of external stimuli (e.g. biotic and abiotic stresses) [57].

Due to the mechanism of action of PWD, terpenoid metabolism is very important in pine tree defense. In *P. pinea* a terpenoid-related protein was also found, namely, a 1-hydroxy-2-methyl-2-(*E*)-butenyl 4-diphosphate synthase (HDS) participant in the 2-C-methyl-D-erythritol 4-phosphate (MEP) pathway. HDS and HD reductase are necessary for resin production and have been already proposed to be important in the physiological response to invasion by the pine wilt disease nematode in *P. densiflora*[58], since PWN progression leads to the cessation of resin flow [2].

One of the main symptoms of PWD is the decrease of photosynthetic rate, which leads to the wilting of leaves. As previous studies of our lab showed, after PWN infestation, the chlorophyll content suffers from a quick decline, specially in *P. pinaster*[59]. Here, a porphobilinogen synthase was identified, a gene directly involved in chlorophyll synthesis [60], that may compensate this decline.

The protein L-isoaspartyl (D-aspartyl) *O*-methyltransferase (PIMT) is commonly present in seed tissues, however its activity is increased under stressful conditions and in *Arabidopsis* it was hypothesised that this protein could be involved in plant stress response [61,62].

Among the up-regulated genes that cannot be directly associated with plant stress response, a ChacC-like protein was identified in *P. pinaster*, as well as a knottin domain, an actin-binding protein and a nitrogen-stress related ammonium transporter; and, in *P. pinea*, a sugar-related phosphate-induced protein with unkown function and an SPX domain, a putative aspartate aminotransferase, a SecY protein and a peptide-N4-(N-acetyl-beta-glucosaminyl) asparagine amidase A (PNGase A). Even though their association with plant disease defense or stress is not yet documented, the current study seems to indicate that they may have a role in the infestation response.

High-throughput sequencing allowed the identification of several candidate genes that may be involved in the response to the PWN. Like in other studies [32], one day after infestation with *B. xylophilus* the plants triggered the expression of genes related to oxidative stress, abiotic or biotic stimulus, plant stress, transcription factors, transport, and secondary metabolites production (Table 3). These genes can be useful targets in genetic transformation and breeding programs that aim at generating maritime pine that is resistant to the PWN.

**Table 3:** General gene function and correspondent genes found between the differentially expressed data

**General function**	**Genes**	**References**
Oxidative stress	Aldo/keto reductase	33
	Multicopper oxidase	45
	2-hydroxyacid dehydrogenase	48
	6-phosphogluconate dehydrogenase	46
	PB1	47
	Cytochrome c	32
	FMN reductase	32
	Malic enzyme	49
	Proline dehydrogenase	50
Defense-related	Sugar related proteins	38, 39, 40
	PAPS reductase	42
	PAR1	36
	Plant Lipid Transfer Protein	37
	Saposin-like	43
	Pectinesterase	52, 53
	PUB-ARM protein	54, 55
	WRKY protein	25, 56
	UBA domain	57
Transcription factors	aminoacyl-tRNA synthetase	25
	ERp29 protein	26
	Translation elongation factor	51
Secondary metabolites production	HMB-PP reductase	30
	HDS	58

### Identification and confirmation of putative defense related genes

Pyrosequencing allowed the identification of 1,423,649 of reads in infested and non infested *P. pinaster* and *P. pinea*, and some of these were expressed at different levels. In order to confirm and compare expression of genes responding to PWN infestation, the expression level of twenty genes previously identified was confirmed by using real time qPCR. A selection was made for genes that were highly represented and other differentially expressed genes that were considered to have particular importance in the defense process.

**Figure 6:**
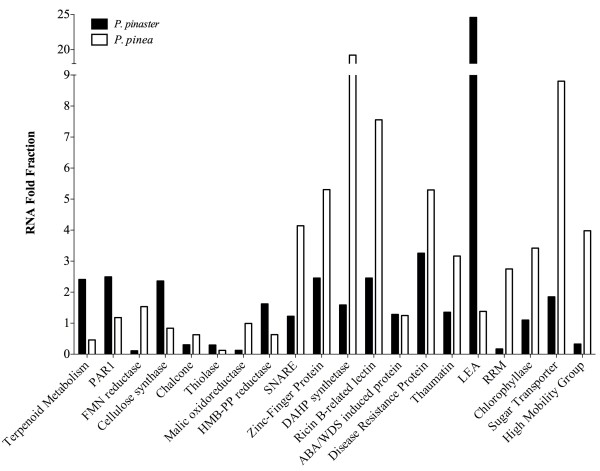
**Quantitative expression of putative defense and stress-related genes to PWN infestation.** The quantitative expression of putative genes from the four pine samples under study was assessed by qPCR. Abundance of transcripts was normalized using the housekeeping gene 18S-rRNA. Milli-Q water was used as control and no amplification was obtained, therefore it is not represented in the figure.

The results confirmed the differential expression of the selected genes, as predicted from the comparative analysis of the transcriptome libraries, suggesting that indeed the data reflects the transcriptional pine profile in response to nematode infection (Figure 6).

From the set of abundantly expressed genes, *P. pinaster* showed higher expression of terpenoid metabolism related proteins, more specifically, HMB-PP reductase and thiolase, which was mentioned before to be important in the plant reaction to nematode infection, defense related PAR1 and cellulose synthase and sugar transport protein.

In *P. pinea* the differential expression of FMN reductase was confirmed. This gene had previously been identified in our laboratory to be involved in the response to PWD [16]. Additionally, the analysis confirmed the differential expression of the malic oxidoreductase (also an antioxidant enzyme) and ricin B-related lectin, that belong to a class of participants in the general defense against a multitude of plant pathogens [44].

Since water stress is directly related to PWD, a protein from a family induced by abcisic acid (ABA) and water deficit stress (WDS) [63] was selected from the set of differentially expressed genes and also a LEA gene [(referred to be related with ABA/WDS induced proteins [63])]. Both had increased expression levels in *P. pinaster* when compared with *P. pinea* (Figure 6). Since oxidative stress is one of the main PWD consequences, a chlorophyllase synthase was also selected, and confirmed to be more expressed by *P. pinaster*. This enzyme catalyzes the hydrolysis of phytol, a oxidative stress related component [64].

As there are reports of phytoalexins showing nematicidal activity in *B. xylophilus*-infested *P. strobus*[65], and since its differential expression in *P. pinea* was detected in the pyrosequencing results, the expression of a chalcone synthase was also analysed. As expected, stone pine (*P. pinea*) expressed this gene two fold higher than maritime pine, which could be an indicator of its lower-susceptibility to *B. xylophilus*.

Other defense response genes detected in the transcriptome that could impose a physical and chemical barrier to nematode progression were the cell wall defense related SNARE protein [66], the abiotic stresses (like drought) related RING zinc finger protein [67], the lignin production related DAHP synthetase [16], and RNA recognition motif connected to protein modification [68]. Up-regulation of genes which constrict nematode progression via increased cell wall strenghtening were also detected in PWN-resistant *P. thumbergii*[15].

The PWD-related thaumatin [32], a disease resistance protein and a gene belonging to a high mobility group family, that in higher plants are required at transcriptional level, specially in the reaction to stress reponses and environmental changes, [69] were also more expressed by *P. pinea*. The genes mentioned above are somehow associated with strong defense responses and, since in nature *P. pinea* trees don’t seem to be as affected by PWD as *P. pinaster*[2], this resistance could be attributed to higher expression of these and other candidate genes in the less susceptible species.

## Conclusions

Since the inoculated samples were expected to be infested with *B. xylophilus* and to have a rich microorganismal community, poly-A RNA was selected as the starting material for the transcriptome library. This should likely eliminate many potential microbial sequences. From the eucaryotic sequences, between 89.1% and 96.5% were plant related. Also, only 1.8% to 12.8% corresponded to *Pinus* spp. sequences, which reflects the scarcity of information available in public databases.

Putative transcripts were sequenced utilizing 454 sequencing technology, which showed that *P. pinaster*, a very susceptible species to the PWN, when infested with *B. xylophilus*, highly expresses genes related to terpenoid secondary metabolism (including some with nematicidal activity), to defense against pathogen attack and to oxidative stress (a common PWD consequence).

On the other hand, *P. pinea* – believed to be less susceptible to this disease – up-regulated transcription regulation related genes, that are needed to activate plant defense responses, and showed higher levels of expression in general of stress response genes such as ricin B-related lectin and disease resistance proteins.

This study establishes a compendium for the understanding of the molecular response of pine trees to PWN, and elucidates the differential defense mechanisms utilized by *P. pinaster* and *P. pinea* against PWN infection.

## Methods

### Plant material and nematode culture

Twenty-eight potted 2-year-old (fourteen *P. pinaster* and fourteen *P. pinea*) trees were used in this study, kept in a climate chamber (Aralab Fitoclima 10000EHF), with relative humidity of 80% and with a photoperiod of 16h day (with photosynthetic active radiation of 490 μmol m^-2^ s^-1^ and temperature of 24–26°C) and 8h night (with temperatures of 19–20°C). Plants were watered every 2 days.

Small, square pieces of Potato Dextrose Agar with *Botrytis cinerea*, grown at 26°C for 7 days, were transferred to test tubes with barley grains previously autoclaved. *B. xylophilus* geographical isolate HF (from Setubal Region, Portugal) was cultured on small squared potato dextrose agar, previously covered with *B. cinerea* mycelium for 7 days at 26°C, placed in test tubes and incubated at 26°C. The multiplied nematodes were extracted using the Baermann funnel technique [70] prior to inoculation. Only nematodes that had been extracted for less than 2 hours were used in the subsequent experiments.

### PWN inoculation and sampling time

The twenty-eight plants were divided in four groups and were inoculated following the method of Futai and Furuno [71]. In brief, a suspension of 1,000 nematodes was pipetted into a small 3–5 cm long longitudinal wound, about 40 cm above soil level. The inoculated wounds were covered with parafilm to prevent drying of the inoculum. The same conditions were applied to the control plants, inoculated with sterile water. Twenty-four hours after inoculation (hai), for each of the seven experimental samples, the entire pine tree stem was cut into small pieces and stored at −80°C until further analysis.

### RNA extraction

Four treatments were studied: *P. pinaster* and *P. pinea* inoculated with *B. xylophilus* strain HF and inoculated with water, as control. A pool of the seven plants from each treatment was made and total RNA was extracted. The extraction was performed according to an optimized method from Provost [72] and the samples were stored at −80°C. RNA integrity and purity was checked by UV-spectrophotometry using a nanophotometer (Implen, Isaza, Portugal) and by fluorimetry.

### cDNA synthesis and pyrosequencing

The total RNA quality was verified on Agilent 2100 Bioanalyzer with the RNA 6000 Pico kit (Agilent Technologies, Waldbronn, Germany) and the quantity assessed by fluorimetry with the Quant-iT RiboGreen RNA kit (Invitrogen, CA, USA). A fraction of 1–2 micrograms of total RNA was used as starting material for cDNA synthesis with the MINT cDNA synthesis kit (Evrogen, Moscow, Russia), a strategy based on the SMART double-stranded cDNA synthesis methodology with amplification of polyA mRNA molecules using a modified template-switching approach that allows the introduction of known adapter sequences to both ends of the first-strand cDNA. The synthesis was done with a modified oligodT containing a restriction site for *Bsg*I. After synthesis, the polyA tails were removed through restriction enzyme digestion to tails and, in that way, minimize the interference of A homopolymers during the 454 sequencing run.

Five hundred nanograms of non-normalized cDNA, quantified by fluorescence, were sequenced in a full plate of 454 GS FLX Titanium according to the standard manufacturer’s instructions (Roche-454 Life Sciences, Brandford, CT, USA) at Biocant (Cantanhede, Portugal).

### Sequence processing, data analysis and functional annotation

Following 454 sequencing, the quality trimming and size selection of reads were determined by the 454 software after which the SMART adaptor sequences were removed from reads using a custom script and the poly-A masked using MIRA, to assure correct assembly of raw sequencing reads [73]. All quality reads were subjected to the MIRA assembler [73] (version 3.2.0), with default parameters.

For some reads, after masking the poly-A, the sequence length was shorter than 40 bp, otherwise the minimum length assumed by the MIRA default parameter settings. The software also disregards all reads that do not match any other read or that belong to the megahub group, i.e. a read that is massively repetitive with respect to other reads. Such reads are considered singlets and were not included in the final assembly result. The entire set of reads used for final assembly was submitted to the NCBI Sequence Read Archive under the accession n° SRA050190.1 (Submission: Control *P. pinea*), SRA050189.1 (Infested *P. pinea*), SRA050188.1 (Control *P. pinaster*) and SRA050187.2 (Infested *P. pinaster*) .

The translation frame of the contigs was determined through queries against the NCBI non redundant protein database using BLASTx with an E-value of 10^-6^ and assessing the best twenty five hits. Contigs without hits were submitted again to BLASTx homology searches against the NCBI nr database with a higher E-value cut- off set at 10^-2^. Sequences with a translation frame identification derived from the two previous searches were used to establish the preferential codon usage in *P. pinaster* and *P. pinea* based on which the software ESTScan [74,75] detected further potential transcripts from the two previous sets of sequences with yet no BLASTx matches. This procedure originated a third set of sequences with putative amino acid translation.

The entire collection of sequences of at least 30 amino acid long, resulting from the BLASTx [76] and the ESTScan procedures, was processed by InterProScan for the prediction of protein domain signatures and Gene Ontology terms. All the results were compiled into a SQL database developed as an information management system. The distribution of sequences into GO categories was calculated at each level and were passed to the parent GO at the top of the broad ontology domains, considering that each single assignment into a GO child was only counted once in the total sum. The positive hits were retrieved and translated into the taxon ID using the information provided by NCBI. In order to obtain a quantitative insight into the taxonomical distribution of the sequences, the different samples were submitted to the MG-RAST server [22]. The MG-RAST provides automated analyses of phylogenetic context, performing the taxonomic evaluation based on the sequence data submitted. The selected parameters for the analysis were: maximum e-value cutoff of 1e-30; minimum percentage identify cutoff of 50%; and minimum alignment length cutoff of 50%. The classification was based in the lowest common ancestor.

### Identification of candidate genes putatively associated with resistance to the PWN

In order to identify the differentially expressed genes, the pyrosequencing results for the infested samples were pooled with the respective control samples and the expression levels of the latter were subtracted, in order to normalize the infested samples.

An interface was implemented in the constructed site with the obtained sequences, to trimm the search in SQL database, using the following algorithm parameters: only sequences with 8 minimum reads were considered and, to ensure the quality of the sequences, the pondered p-value was of 5e^-05^. These strict parameters were established to limit the search only to the most represented genes.

After the application of this algorithm, all reads from the same sequences were grouped and the genes with unknown function were removed from the analysis. A ratio between the normalized infested samples was calculated, with which all sequences with a ratio inferior to 1 were excluded and hits with ratios higher than 1 were considered to be overexpressed for the numerator sample.

### Confirmation of differential expression of candidate genes

Candidate genes were selected following queries performed to the pyrosequencing database using distinct search descriptors based on BLAST hit descriptions, GO descriptions, Interpro descriptions, GO and Interpro identification numbers. Queries were aimed at the identification of genes described in the literature as being related to immunity and inflammatory reactions.

The same plant material that was used for the pyrosequencing experiment was used for quantitative real-time PCR (qPCR) to assess and quantify the relative expression of the candidate genes. Primers targeting the resistance candidate genes were designed using the OligoPerfect™ Designer tool from Invitrogen, specifying an expected PCR product of 200–300 bp and primer annealing temperatures between 56°C and 58°C. The sequences are presented in Table 4. qPCR reactions were performed on a Chromo4 thermocycler (Bio-Rad, CA, USA). Amplifications were carried out using 1.25 μM of the specific primers and mixed to 12.5 μL of 2×PCR iQ SYBR Green Supermix (Bio-Rad) and 100 ng of cDNA in a final volume of 25 μl. Three replicates were performed for each gene tested in qPCR reactions, as well as for controls. Melt curves profiles were analyzed for each gene tested. The 18S rRNA gene was used as the housekeeping gene and for normalization of expression of gene of interest or defense-related target genes. The comparative CT method (ΔΔCT) for the relative quantification of gene expression was used for assessing the normalized expression value of defense-related genes using the 18S rRNA as the control transcript (Opticon Monitor 3 Software, Bio-Rad). Data were transferred to Excel files and plotted as histograms of normalized fold expression of target genes.

**Table 4:** Forward and reverse primer sequences used in quantitative real time PCR analyses

**Cadidate gene**	**5’-3’ forward primer**	**5’-3’ reverse primer**
rRNA 18S	TTAGGCCATGGAGGTTTGAG	GAGTTGATGACACGCGCTTA
Terpenoid metabolism	TCCTGATCGCTTTCATCCTT	AGATGGTTCATGGGGAACTG
PAR1	CACAGACGGGGCAAGTAGAT	AGAGGATGACAGTGGGGATG
FMN reductase	AGGTTCCGGAAACACTTCCT	CAATTGCTGAGTTCGCCATA
Cellulose synthase	AAGCCCCTCCCTCTCAAATA	TCATCATCAAGCACACAGCA
Chalcone synthase	TCCCACATCCAATCCTTCTC	TTCCAGCAGTTCGGAATCTC
Thiolase	CCCATTCCTTTGCCTCAATA	CGGCTCTAGCCATACCAAAA
Malic oxireductase	GTTTGTTTAGACGGCCGAGA	AGGAAGCACCCTTTGAGGAT
HMB-PP reductase	CAATGCAACTGAAGGAGCAA	TTGGGAGCGAACATCCTATC
SNARE	GGGTGGGCTCTTTGGATAAT	TTAACTGCAACCCGTTTTCC
RING-HC Zinc Finger	AAGCCACAAACCACGAAATC	GAGATTGCCCTAACCGTGAA
DAHP synthetase	CCACCAATGCATTCTGTCAC	CCCTTTGACGCAATAAGAGC
Ricin B related lectin	GCAGCCAAGAAAAACTCTGG	ATTGGGTGCTTCACAAGGAG
ABA/WDS induced prt	AAAAGCGACAAGCGTAAGGA	CACGGCCAAGCTTAAAAGAG
Disease Resistance Prt	GGTTGAATGTGCCCTCACTT	GGGAAGCTTTAGGCTCGACT
Thaumatin	CGGGggATACTCAGACTTGA	GAATTGAACGGTCCACGACT
LEA	GAGGATCACTTTGGCGAGAC	AGTCTACAGCCGCACGAACT
RNA recognition motif	GACTTTTCCTGGTGCTCTGC	CAGGTATGCCCAGACCAGTT
Chlorophyllase	GTAGGAGGAATTGGCGATCA	AATCTTGGATCCACCACAGC
Sugar transporter	CATGTTGATTATCGCGTTGG	AACCCTACTGCCATTGTTGC
High Mobility Group	CGCTTTCCAATAGGCTTGTC	TGCGTTTCACTCTGTTACGG

## Competing interests

The authors declare that they have no competing interests.

## Authors’ contributions

CSS carried out sample preparation for the analysis, the gene confirmation experiments and drafted the manuscript. MP developed the pipe-line analysis for all functional annotation and developed the database. CE helped conceive the study, oversaw sequencing and participated in the critical review of the manuscript. AIS contributed to the bioinformatic analysis of the data. MWV conceived the study, participated in its design and coordination and helped to draft the manuscript. All authors read and approved the final manuscript.
